# CPD-1 can compensate for EGL-21 to process neuropeptides

**DOI:** 10.17912/micropub.biology.001965

**Published:** 2026-01-06

**Authors:** David C Khawand, Amy K Clippinger, Michael Ailion

**Affiliations:** 1 Department of Biochemistry, University of Washington, Seattle, WA USA

## Abstract

Carboxypeptidase D has been thought to process neuropeptides, though its role has not been fully characterized. Since specific neuropeptides regulate the defecation motor program of *C. elegans* , we used genetic analysis to determine how loss of the worm carboxypeptidase D ortholog CPD-1 affects defecation. We found that *cpd-1* mutants do not have defecation defects but enhance the defecation defects of *egl-21* mutants lacking carboxypeptidase E, a major neuropeptide processing enzyme. We also found that CPD-1 acts in intestinal cells and possibly GABAergic neurons to promote defecation. These results suggest that CPD-1 can process neuropeptides, specifically in the absence of EGL-21 .

**Figure 1. CPD-1 expression in intestine and GABAergic neurons rescues Exp and aBoc defects in egl-21 and cpd-1; egl-21 worms:**
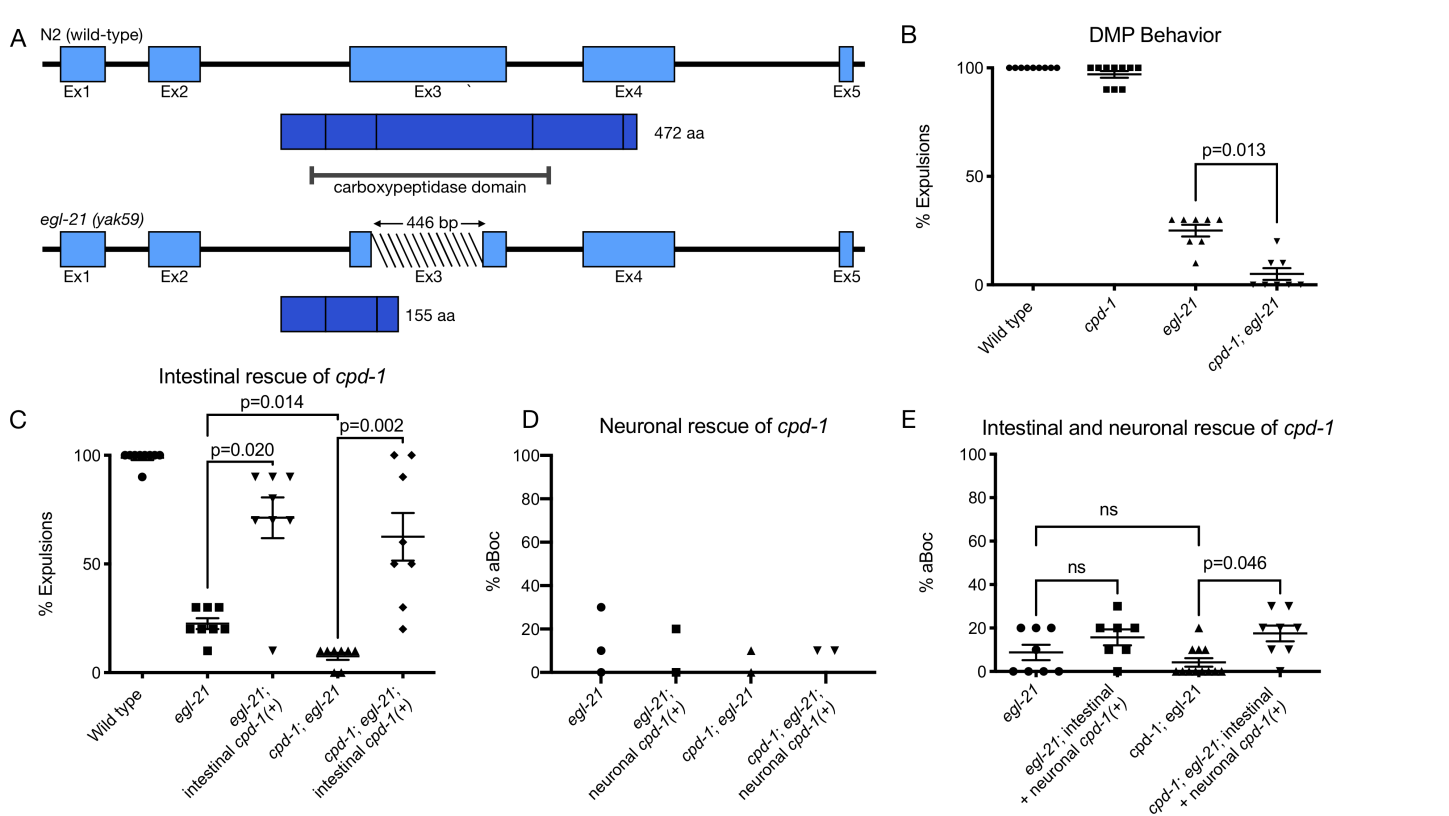
(A) The new *egl-21* allele *yak59* has a deletion in the carboxypeptidase domain and leads to an early stop. (B) Exp percentages for N2 (n=9), *cpd-1* (n=10), *egl-21* (n=8), and *cpd-1 ; egl-21* (n=8) worms. (C) Exp percentages for N2 (n=9), *egl-21* (8), and *cpd-1 ; egl-21* (n=8) worms alongside mutants expressing *cpd-1 (+)* in the intestine (n=8). (D) aBoc percentages for *egl-21* (n=3) and *cpd-1 ; egl-21* (n=2) worms alongside mutants expressing *cpd-1 (+)* in GABAergic neurons (n=2). (E) aBoc percentages for *egl-21 (n=8)* , and *cpd-1 ; egl-21* (n=12) worms alongside mutants *egl-21* and *cpd-1 ; egl-21* expressing *cpd-1 (+)* in the intestine and GABAergic neurons (n = 7 and 8, respectively).

## Description

Neuropeptides are derived from larger precursor proteins that undergo processing by proprotein convertase and carboxypeptidase enzymes to achieve their functional forms. Carboxypeptidase E (CPE) is thought to be the major carboxypeptidase contributing to neuropeptide processing (Ji et al., 2017), but humans and rodents have an additional carboxypeptidase, carboxypeptidase D (CPD). CPE and CPD have similar proteolytic activity against C-terminal basic amino acids (Song & Fricker, 1995). As mice lacking CPE are able to produce some fully processed hormones and neuropeptides, it has been suggested that CPD can partially compensate for the role of CPE (Chen et al., 2023; Dong et al., 1999; Ji et al., 2017). The nematode *C. elegans* has an ortholog of mammalian CPE, EGL-21 (Jacob & Kaplan, 2003), and two homologs of mammalian CPD, CPD-1 and CPD-2 , which have not been previously characterized. Human, rat, mouse, and duck CPD contain three carboxypeptidase domains followed by a transmembrane domain and a highly conserved cytosolic tail (Sidyelyeva & Fricker, 2002). In *C. elegans* , both CPD-1 and CPD-2 have three carboxypeptidase domains, but only CPD-1 is predicted to have a transmembrane domain and a cytosolic tail and therefore is the most similar to vertebrate CPD.

&nbsp;

To investigate the role of CPD in neuropeptide processing in *C. elegans* , we focused on the defecation motor program (DMP), a stereotyped rhythmic behavior consisting of three steps: a posterior body contraction (pBoc), an anterior body contraction (aBoc), and finally an expulsion (Exp) step (Thomas, 1990). Previous studies identified neuropeptides involved in the DMP, such as NLP-40 , which is essential for the Exp step and regulates the aBoc step to a lesser degree (Choi et al., 2023; Wang et al., 2013). Mutations in neuropeptide processing enzymes also lead to defects in DMP, though which step is affected and the extent of impairment varies. For example, the carboxypeptidase EGL-21 and the pro-protein convertase AEX-5 regulate the aBoc and Exp steps, while the pro-protein convertase EGL-3 regulates the aBoc step only (Mahoney et al., 2008; Nagy et al., 2015). It remains unclear which processing enzymes work on specific peptides.

&nbsp;

In this study, we used a new allele of *egl-21* , *yak59* , which has a deletion that leads to an early stop and is expected to be a null mutation ( Figure 1A ). *egl-21 (yak59)* mutants have about 25% expulsions ( Figure 1B ), similar to that observed in other *egl-21* alleles (Hao et al., 2012; Nagy et al., 2015). Worms lacking *cpd-1* have a normal level of expulsions ( Figure 1B ). To determine whether CPD-1 is responsible for the residual expulsions observed in *egl-21* mutants, we assayed *cpd-1 ; egl-21* double mutants and found that they had very few expulsions ( Figure 1B ), suggesting that CPD-1 contributes to peptide processing in the absence of EGL-21 .

&nbsp;

The neuropeptide NLP-40 serves as the main driver of the Exp step (Wang et al., 2013). NLP-40 processing is impaired in *egl-21* mutant worms (Husson et al., 2007), likely explaining their Exp defect. Since NLP-40 processing occurs in the intestine, we tested whether CPD-1 acts in the intestine. We found that intestinal-specific expression of *cpd-1* strongly rescued the Exp defects of both *egl-21* mutants and *cpd-1 ; egl-21* double mutants ( Figure 1C ). These data indicate that CPD-1 can process NLP-40 in the intestine, and that EGL-21 is not required when CPD-1 is overexpressed. We also attempted to rescue *egl-21* and *cpd-1* ; *egl-21* mutants with intestinal *egl-21* (+), but found that extrachromosomal arrays of *egl-21* driven by the *vha-6* promoter caused a constipation phenotype in N2 animals, indicating that *egl-21* overexpression in the intestine impairs defecation.

&nbsp;

Neuropeptides released from GABAergic neurons, such as FLP-22 , are also known to regulate the DMP, in particular the aBoc step (Choi et al., 2023). Since EGL-21 is known to process FLP-22 (Husson et al., 2007), we hypothesized that CPD-1 might also function in GABAergic neurons to process FLP-22 . Mutant *egl-21* worms have been reported to be aBoc deficient (Nagy et al., 2015), and in agreement, we found that aBocs occur in *egl-21* mutants at a low frequency of about 9% ( Figure 1E ). *cpd-1 ; egl-21* double mutants had an even lower aBoc frequency (~ 4%, Figure 1E ). The difference in aBoc percentage between *egl-21* and *cpd-1 ; egl-21* mutants was not statistically significant, but we note that this experiment was underpowered for detecting this small difference. NLP-40 is required for normal aBoc frequency (Wang et al., 2013), and given that our data suggest that CPD-1 contributes to NLP-40 processing ( Figure 1B & C), it is expected that *cpd-1 ; egl-21* double mutants should have a more severe aBoc defect than *egl-21* mutants, whether or not CPD-1 also contributes to the processing of FLP-22 .

&nbsp;

Expressing *cpd-1* specifically in GABAergic neurons failed to rescue *egl-21* or *cpd-1 ; egl-21* double mutants ( Figure 1D ), presumably because processing of NLP-40 in the intestine is still impaired, and NLP-40 is required for the downstream release of neuropeptides from GABAergic neurons (Choi et al., 2023). Thus, we expressed *cpd-1* in both the intestine and GABAergic neurons of *egl-21* and *cpd-1 ; egl-21* mutant worms, and found there were increases in aBoc frequency compared to *egl-21* and *cpd-1 ; egl-21* mutants ( Figure 1E ). However, this rescue was only statistically significant for the *cpd-1 ; egl-21* double mutant. The incomplete rescue of the aBoc phenotype may be due to residual defects in NLP-40 processing in the intestine; expressing *cpd-1* in the intestine in *egl-21* and *cpd-1 ; egl-21* mutants did not rescue Exp to 100% ( Figure 1C ), indicating that NLP-40 processing is not returned to normal levels and thus the downstream neuronal release of neuropeptides that depend on NLP-40 might also not be returned to normal levels, independently of the extent of neuropeptide processing in neurons. The best experiment to determine whether CPD-1 acts in neuropeptide processing in GABAergic neurons would be to rescue *egl-21* in the intestine and then rescue *cpd-1* in the neurons, because this would specifically assay for *cpd-1* function in neurons when signaling from the intestine is normal. But overexpressing *egl-21* in intestine caused a constipated phenotype on its own, so this experiment would require a single-copy *vha-6 p:: egl-21* transgene which we did not obtain.

&nbsp;

Taken together, these experiments demonstrate that CPD-1 can compensate for EGL-21 in the processing of neuropeptides in the intestine and possibly in GABAergic neurons. These results are consistent with previous observations in mammalian organs like the brain, pancreas, and intestine, where CPD was identified as a possible enzyme that compensates for the role of CPE in peptide processing (Chen et al., 2023; Dong et al., 1999; Ji et al., 2017).

## Methods

***Strain maintenance***

*C. elegans* worms were maintained at room temperature (~22°C) on NGM agar plates seeded with lawns of OP50 bacteria.

*&nbsp;*

***Plasmid and Strain Construction***

A complete list of plasmids is provided in Table M1. Constructs were made utilizing the three-slot multisite Gateway system (Invitrogen). The *cpd-1* cDNA was cloned from a cDNA library generated from N2 , and has a single 180 bp intron between exon 10 and exon 11. For intestinal expression of *cpd-1* and *egl-21* , the intestinal promoter *vha-6* , *cpd-1* cDNA or *egl-21* genomic DNA, and *let-858* 3'UTR were cloned into the pCFJ150 destination vector. For neuronal expression of *cpd-1* , the GABAergic neuronal promoter *unc-47p* , *cpd-1* cDNA, and GFP operon were cloned into the pCFJ150 destination vector.

&nbsp;

***Tissue specific cpd-1 expression and rescue experiments***

Extrachromosomal arrays were generated for intestinal expression by injecting pDK1 or pDK2 with pMA122, pCFJ601, and pPD97/98 into *unc-119* worms ( EG6699 or EG8079 , respectively), and for GABAergic neuron expression by injecting pDK3 and pCFJ90 into N2 worms. After obtaining stable arrays of *cpd-1 (+)* intestinal expression ( *yakEx268* , strain XZ2582) and GABAergic expression ( *yakEx277* , XZ2665) these strains were crossed with *cpd-1 ; egl-21* (XZ2605) to generate *cpd-1 ; egl-21 ; yakEx268* (XZ2628) and *egl-21 ; yakEx268* (XZ2627), *cpd-1 ; egl-21 ; yakEx277* (XZ2667) and *egl-21 ; yakEx277* (XZ2666). Then double mutant strains containing each array (XZ2628 and XZ2667) were crossed to generate *cpd-1 ; egl-21 ; yakEx268; yakEx277* &nbsp;(XZ2669) and *egl-21 ; yakEx268; yakEx277* (XZ2668).

&nbsp;

**DMP Cycle Measurements**

All defecation data were collected using the computer program Etho (version 1.2.2) (Thomas, 2007). The start of each DMP cycle was marked after observing a pBoc. A total of 10 DMP cycles were observed for each animal, and either the time of the expulsion or the aBoc step was recorded for Exp or aBoc measurements, respectively.

&nbsp;

**Statistical Analysis**

Because the data we acquired had small sample sizes and were non-continuous, we performed statistical analysis using non-parametric permutational analysis of variance (PERMANOVA) followed by post hoc pairwise permutation t-tests with Bonferroni correction (10,000 permutations for Figure 1B & C and 100,000 permutations for Figure 1E ). Statistical analysis was done in R (version 4.2.3) using the functions perm.anova and pairwise.perm.t.test from the package ‘RVAideMemoire' (version 0.9-83-7) (Herve, 2025).

## Reagents

**Table M1.**

**Table:** 

Name	Plasmid	Source (if not Ailion Lab)
Gateway Destination Vectors		
pCFJ150&nbsp;&nbsp;&nbsp;&nbsp;	Gateway destination vector for Mos site *ttTi5605*	Jorgensen Lab
Gateway Entry Clones		
pADA-126	*let-858* 3'UTR [2-3]	Jorgensen Lab
pCFJ326&nbsp;&nbsp;&nbsp;	GFP operon [2-3] ( *tbb-2* 3'UTR operon GFP::H2B *cye-1* 3'UTR)	Jorgensen Lab
pJB1&nbsp;	*cpd-1* cDNA [1-2]	
pLC110&nbsp;&nbsp;	*vha-6 p* no ATG [4-1]	
pMH522&nbsp;&nbsp;&nbsp;&nbsp;	*unc-47p* [4-1]	Jorgensen Lab
pSD20&nbsp;&nbsp;	*egl-21* genomic DNA [1-2]	Jorgensen Lab
Expression Constructs		
pDK1&nbsp;&nbsp;	*vha-6 p:: cpd-1 cDNA:: let-858* 3'UTR	
pDK2 *&nbsp;&nbsp;&nbsp;&nbsp;&nbsp;*	*vha-6 p:: egl-21 genomic DNA:: let-858 3'UTR *	
pDK3&nbsp;&nbsp;&nbsp;&nbsp;&nbsp;&nbsp;	* unc-47p:: cpd-1 cDNA::GFP operon *	
pMA122	* hsp-16.41p:: peel-1 cDNA:: tbb-2* 3'UTR	
pCFJ601&nbsp;&nbsp;	*eft-3p::* Mos1 transposase	Jorgensen Lab
pPD97/98	*unc-122p::GFP* (cc *::GFP* )	Jorgensen Lab
pBS_SK&nbsp;	pBluescript	Chalfie Lab
pCFJ90&nbsp;&nbsp;&nbsp;&nbsp;	*myo-2p::mCherry*	Jorgensen Lab

**Table M2.**

**Table:** 

Strain Name	Genotype	Source (if not Ailion Lab)
EG6699 &nbsp;&nbsp;	*ttTi5605 II ; unc-119 ( ed3 ) III *	Jorgensen Lab
EG8079 &nbsp;	*oxTi179 [ ttTi5605 + NeoR(+) + unc-18 (+)] II ; unc-119 ( ed3 ) III *	Jorgensen Lab
FX3451&nbsp;&nbsp;	*cpd-1 ( tm3451 ) I *	National BioResource Project (NBRP)
N2 &nbsp; &nbsp;	Bristol isolate, standard lab wild type	Jorgensen Lab
XZ59	*egl-21 (yak59) IV* outcrossed 2X	
XZ2582&nbsp;&nbsp;	* &nbsp; ttTi5605 II ; unc-119 ( ed3 ) III ; yakEx268[ vha-6 p:: cpd-1 cDNA, hs:: peel-1 ,* &nbsp;&nbsp;&nbsp;&nbsp;&nbsp;&nbsp;&nbsp;&nbsp;&nbsp;&nbsp;&nbsp;&nbsp;&nbsp;&nbsp;&nbsp;&nbsp;&nbsp;&nbsp;&nbsp;&nbsp;&nbsp;&nbsp;&nbsp;&nbsp;&nbsp;&nbsp;&nbsp;&nbsp;&nbsp;&nbsp;&nbsp;&nbsp;&nbsp; *eft-3p::Mosase, cc::GFP]*	
XZ2585	*oxTi179 [ ttTi5605 + NeoR(+) + unc-18 (+)] II; unc-119 ( ed3 ) III; yakEx271[ vha-6 p:: egl-21 (+), hs:: peel-1 , eft-3p::Mosase, cc::GFP] *	
XZ2586	*oxTi179 [ ttTi5605 + NeoR(+) + &nbsp; unc-18 (+)] II; unc-119 ( ed3 ) III;* *yakEx272[ vha-6 p:: egl-21 (+), hs::peel-1, eft-3p::Mosase, cc::GFP] *	
XZ2591&nbsp;&nbsp;&nbsp;&nbsp;&nbsp;	*cpd-1 ( tm3451 ) I* outcrossed 3X	
XZ2605&nbsp;	*cpd-1 ( tm3451 ) I; egl-21 (yak59) IV *	
XZ2627&nbsp;&nbsp;&nbsp;	*egl-21 (yak59) IV ; yakEx268[ vha-6 p:: cpd-1 cDNA, hs:: peel-1 , eft-3p::Mosase, cc::GFP]&nbsp; *	
XZ2628&nbsp;&nbsp;&nbsp;	*cpd-1 ( tm3451 ) I ; egl-21 (yak59) IV ; yakEx268[ vha-6 p:: cpd-1 cDNA, hs:: peel-1 , eft-3p::Mosase, cc::GFP] *	
XZ2665	* yakEx277[unc-47p:: cpd-1 cDNA::GFP operon, myo-2p::mCherry]&nbsp;&nbsp;&nbsp; *	
XZ2666&nbsp;&nbsp;	*egl-21 (yak59) IV ;&nbsp; yakEx277[unc-47p:: cpd-1 cDNA::GFP operon, myo-2p::mCherry]&nbsp;&nbsp;&nbsp; *	
XZ2667&nbsp;	*cpd-1 ( tm3451 ) I ; egl-21 (yak59) IV ; yakEx277[unc-47p:: cpd-1 cDNA::GFP operon, myo-2p::mCherry] *	
XZ2668&nbsp;&nbsp;	*egl-21 (yak59) IV ; yakEx268[ vha-6 p:: cpd-1 cDNA, hs:: peel-1 , eft-3p::Mosase, cc::GFP] ; yakEx277[unc-47p:: cpd-1 cDNA::GFP operon, myo-2p::mCherry]&nbsp;&nbsp;&nbsp;&nbsp;&nbsp;&nbsp;&nbsp;&nbsp; *	
XZ2669	*cpd-1 ( tm3451 ) I ; egl-21 (yak59) IV ; yakEx268[ vha-6 p:: cpd-1 cDNA, hs:: peel-1 , eft-3p::Mosase, cc::GFP] ; yakEx277[unc-47p:: cpd-1 cDNA::GFP operon, myo-2p::mCherry]&nbsp;&nbsp; *	

*&nbsp;&nbsp;&nbsp;&nbsp;&nbsp;*
